# A Rationally Designed Reversible ‘Turn-Off’ Sensor for Glutathione

**DOI:** 10.3390/bios7030036

**Published:** 2017-09-06

**Authors:** Sabrina Heng, Xiaozhou Zhang, Jinxin Pei, Andrew D. Abell

**Affiliations:** 1 ARC Centre of Excellence for Nanoscale BioPhotonics, Institute of Photonics and Advanced Sensing, Department of Chemistry, School of Physical Sciences, The University of Adelaide, Adelaide SA 5005, Australia; xiao.z.zhang@adelaide.edu.au; 2 Discipline of Physiology, Faculty of Health Sciences, The University of Adelaide, Adelaide SA 5005, Australia; jinxin.pei@adelaide.edu.au

**Keywords:** reversible sensor, photoswitch, spiropyran, fluorescent sensor, reduced glutathione, intracellular sensing, turn-off sensor

## Abstract

γ-Glutamyl-cysteinyl-glycine (GSH) plays a critical role in maintaining redox homeostasis in biological systems and a decrease in its cellular levels is associated with diseases. Existing fluorescence-based chemosensors for GSH acts as irreversible reaction-based probes that exhibit a maximum fluorescence (‘turn-on’) once the reaction is complete, regardless of the actual concentration of GSH. A reversible, reaction-based ‘turn-off’ probe (**1**) is reported here to sense the decreasing levels of GSH, a situation known to occur at the onset of various diseases. The more fluorescent merocyanine (MC) isomer of **1** exists in aqueous solution and this reacts with GSH to induce formation of the ring-closed spiropyran (SP) isomer, with a measurable decrease in absorbance and fluorescence (‘turn-off’). Sensor **1** has good aqueous solubility and shows an excellent selectivity for GSH over other biologically relevant metal ions and aminothiol analytes. The sensor permeates HEK 293 cells and an increase in fluorescence is observed on adding buthionine sulfoximine, an inhibitor of GSH synthesis.

## 1. Introduction

γ-Glutamyl-cysteinyl-glycine (GSH) is the most abundant thiol in biological systems [1,2], with a central role in critical cellular functions including redox regulation and cellular signalling [3,4,5,6,7]. Current strategies for its sensing include high performance liquid chromatography [8], gas chromatography-mass spectrometry [9], electro-chemical assays [10]**,** and spectroscopic detection using fluorescent chemosensors [11,12,13]. Typical fluorescent ‘turn on’ chemosensors function as reaction-based probes, binding to GSH irreversibly with a high sensitivity and selectivity [14,15]. Here, an increase in GSH levels is accompanied by an increase in fluorescence [16,17]. This is fine for healthy cells that possess base-line millimolar concentrations of GSH [18]. However, many diseases including Parkinson’s [19,20], cancer [21,22], heart diseases [23]**,** and Alzheimer’s [24] are indicated by a decrease in GSH levels. In this case, a ‘turn on’ sensor would result in reduced fluorescence relative to healthy cells. An important advance would come from the development of a sensor that is measurably turned off by GSH, and back on by a lower level of GSH. This would then provide an opportunity to sense reduced GSH levels during the onset of important diseases [25].

**Figure 1:**
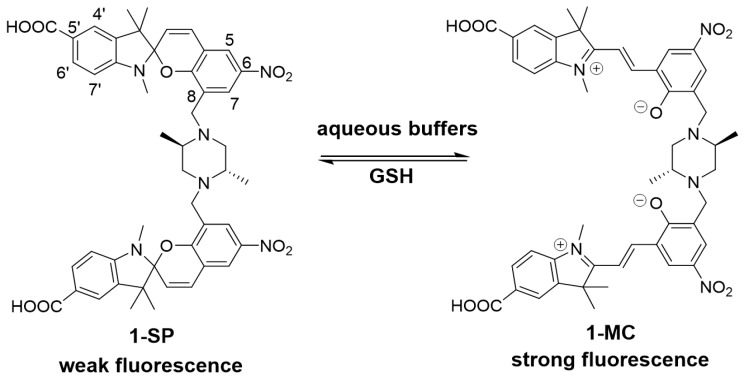
Spiropyran (SP) and merocyanine (MC) isomers of sensor **1**. The ring-closed spiropyran (**1-SP**) is weakly fluorescent and the ring-opened merocyanine (**1-MC**) more fluorescent [30].

A spiropyran-based ‘turn-on’ sensor that selectively and reversibly detects GSH in cells has been recently reported [26,27]. This sensor contains two spiropyran units linked by an unsubstituted piperazine, and exists as the ring closed spiropyran isomer (SP) in solution. Exposure to GSH switches the sensor on through formation of the fluorescent merocyanine (MC) isomer, which is proposed to bind non-covalently to GSH [26]. The sensor shows excellent selectivity for GSH over other intracellular thiol-containing species [26]. Multiple measurements on a single sample are possible without the need to change the sensor [28,29]. Here, we report a new reversible ‘turn-off’ spiropyran-based GSH sensor (**1**, Figure 1) where the fluorescence and absorbance are inversely proportional to the level of GSH. The sensor has good water solubility and biocompatibility and is suitable for intracellular sensing.

## 2. Materials and Methods

### 2.1. General Information

Unless otherwise indicated, all starting materials, chemicals, and anhydrous solvents were purchased from Sigma Aldrich (NSW, Australia) and were used without further purification. ^1^H and ^13^C-NMR spectra were recorded on a Varian 500 MHz or a Varian Inova 600 MHz instruments in the indicated solvents. Chemical shifts are reported in ppm (δ). Signals are reported as s (singlet), br s (broad singlet), d (doublet), dd (doublet of doublets), t (triplet), or m (multiplet). High resolution mass spectra were collected using an LTQ Orbitrap XL ETD using flow injection, with a flow rate of 5 μL/min. Where indicated compounds were analyzed and purified by reverse phase HPLC, using an HP 1100 LC system equipped with a Phenomenex C-18 column (250 × 4.6 mm) for analytical traces and a Gilson GX-Prep HPLC system equipped with a Phenomenex C18 column (250 × 21.2 mm). H_2_O and MeCN solutions were used as aqueous and organic buffers. All absorbance and fluorescence spectra were collected on a Synergy H4 Hybrid Multimode Microplate Reader using black-walled clear bottom 96-well plates. Data was processed using Microsoft Excel 2016 and all graphs were generated using GraphPad Prism 7 software. A mercury lamp (365 nm) was used as the UV light source in photoswitching experiments.

### 2.2. Chemical Syntheses

#### 2.2.1. 3,3′-(((2S,5R)-2,5-Dimethylpiperazine-1,4-diyl)bis(methylene))bis(2-hydroxy-5-nitrobenzaldehyde) (**4a**)

To a solution of compound **2a** (250 mg, 1.16 mmol) in anhydrous THF (25 mL) was added compound **3** (66 mg, 0.58 mmol) in one portion. The mixture was stirred under N_2_ while triethylamine (234 mg, 2.32 mmol) was added dropwise. The reaction mixture was heated to reflux for 18 h under N_2_. The mixture was cooled to r.t. and the precipitate was collected and washed with MeOH (5 mL) under vacuum to yield the compound **4a** as a yellow solid (254 mg, 93%). ^1^H-NMR (500 MHz, DMSO-*d*_6_) δ 10.25 (s, 2H), 7.37 (d, *J* = 8.8 Hz, 2H), 7.29 (d, *J* = 8.8 Hz, 2H), 4.26 (d, *J* = 15.0 Hz, 2H), 3.46 (d, *J* = 15.0 Hz, 2H), 2.84 (d, *J* = 10.7 Hz, 2H), 2.58 (br s, 2H), 2.17 (t, *J* = 11.1 Hz, 2H), 1.78–1.72 (m, 2H), 1.08 (d, *J* = 6.3 Hz, 6H). ^13^C-NMR (126 MHz, DMSO-*d*_6_) δ 193.3, 160.5, 158.9, 157.1, 130.6, 125.6, 114.9, 70.1, 58.8, 56.8, 28.2. HRMS (ESI) found [M + H]^+^ 473.1663, C_22_H_25_N_4_O_8_^+^ requires 473.1672.

#### 2.2.2. 3,3′-(((2S,5R)-2,5-Dimethylpiperazine-1,4-diyl)bis(methylene))bis(2-hydroxy-5-methylbenzaldehyde) (**4b**)

To a solution of compound **2b** (485 mg, 2.63 mmol) in anhydrous THF (10 mL) was added to compound **3** (150 mg, 1.32 mmol) in one portion. The mixture was stirred under N_2_ while triethylamine (530 mg, 5.25 mmol) was added dropwise. The reaction mixture was heated to reflux for 18 h under N_2_. The mixture was cooled to r.t. and the volatiles were removed in vacuo. The crude mixture was purified by flash column chromatography (DCM:MeOH 98:2) to yield the compound **4b** as a yellow solid (200 mg, 37%). ^1^H-NMR (500 MHz, CDCl_3_) δ 10.25 (s, 1H), 7.43 (s, 1H), 7.17 (s, 1H), 4.29 (d, *J* = 14.1 Hz, 1H), 3.25 (d, *J* = 14.1 Hz, 1H), 2.86 (d, *J* = 12.0 Hz, 1H), 2.58 (br s, 1H), 2.31 (s, 3H), 2.13 (t, *J* = 11.1 Hz, 1H), 1.17 (d, *J* = 6.2 Hz, 3H). ^13^C-NMR (126 MHz, CDCl_3_) δ 190.4, 156.9, 134.7, 127.2, 126.6, 122.1, 120.4, 114.5, 57.5, 54.4, 52.4, 48.9, 18.4. HRMS (ESI) found [M]^+^ 410.2202, C_24_H_30_N_2_O_4_^+^ requires 410.2206.

#### 2.2.3. 8,8′′-(((2R,5S)-2,5-Dimethylpiperazine-1,4-diyl)bis(methylene))bis(1′,3′,3′-trimethyl-6-nitrospiro[chromene-2,2′-indoline]-5′-carboxylic acid) (**1**)

To a solution of compound **4a** (100 mg, 0.21 mmol) in anhydrous MeCN (6 mL) was added to compound **5** [31] (115 mg, 0.53 mmol) and piperidine (49 mg, 0.57 mmol). The mixture was heated to reflux for 18 h under N_2_. The mixture was cooled to r.t. and the precipitate was washed with MeCN (5 mL) and collected by vacuum filtration. This was then purified by reverse-phase HPLC to give compound **1** as a pale brown solid (32 mg, 18%). ^1^H-NMR (500 MHz, DMSO-*d*_6_) δ 8.28–8.07 (m, 2H), 8.02–7.89 (m, 2H), 7.80–7.74 (m, 2H), 7.69 (d, *J* = 5.7 Hz, 2H), 7.25 (d, *J* = 10.8 Hz, 2H), 6.69–6.55 (m, 2H), 6.01 (d, *J* = 10.3 Hz, 2H), 3.74–3.58 (m, 2H), 2.82–2.70 (m, 2H), 2.68 (d, *J* = 15.4 Hz, 6H), 2.29–2.13 (m, 2H), 1.96–1.86 (m, 2H), 1.57–1.45 (m, 2H), 1.26 (s, 6H), 1.15 (s, 6H), 0.46–0.33 (m, 6H). ^13^C-NMR (126 MHz, DMSO-*d*_6_) δ 167.9, 155.4, 151.6, 140.9, 136.4, 131.3, 130.1, 129.0, 123.3, 122.8, 122.1, 121.6, 118.6, 107.3, 106.8, 106.2, 72.7, 60.7, 57.0, 51.7, 28.8, 25.9, 20.0. HRMS (ESI) found [M − H]^−^ 869.3503, C_48_H_49_N_6_O_10_^−^ requires 869.3510.

#### 2.2.4. 8,8′′-(((2R,5S)-2,5-Dimethylpiperazine-1,4-diyl)bis(methylene))bis(1′,3′,3′,6-tetramethylspiro[chromene-2,2′-indoline]-5′-carboxylic acid) (**6**)

To a solution of compound **4b** (100 mg, 0.21 mmol) in anhydrous EtOH (6 mL) was added to compound **5** [31] (115 mg, 0.53 mmol) and triethylamine (58 mg, 0.57 mmol). The mixture was heated to reflux for 18 h under N_2_. The mixture was cooled to r.t. and the solvent was removed in vacuo and the resultant crude mixture was purified by reverse-phase HPLC to give compound **6** as a brown solid (24 mg, 14%). ^1^H-NMR (500 MHz, DMSO-*d*_6_) δ 7.74 (d, *J* = 8.2 Hz, 1H), 7.64 (s, 1H), 6.97 (d, *J* = 10.1 Hz, 1H), 6.88 (d, *J* = 8.2 Hz, 1H), 6.80 (s, 1H), 6.60–6.37 (m, 1H), 5.75 (d, *J* = 10.1 Hz, 1H), 3.68 (d, *J* = 12.5 Hz, 1H), 3.56 (d, *J* = 12.5 Hz, 1H), 2.65 (s, 3H), 2.60 (d, *J* = 7.6 Hz, 1H), 2.19 (s, 3H), 1.95–1.67 (m, 1H), 1.47–1.29 (m, 1H), 1.24 (s, 3H), 1.11 (d, *J* = 9.6 Hz, 3H), 0.35 (s, 3H). ^13^C-NMR (151 MHz, DMSO-*d*_6_) δ 170.5, 154.0, 153.3, 139.2, 136.9, 133.9, 132.8, 132.4, 126.0, 124.3, 122.0, 121.8, 118.0, 108.8, 107.8, 75.4, 63.3, 56.2, 54.6, 53.8, 31.5, 28.6, 25.4, 23.9, 22.9. HRMS (ESI) found [M + H]^+^ 809.4299, C_50_H_57_N_4_O_6_^+^ requires 809.4278.

### 2.3. Spectroscopic Analyses of Sensors ***1*** and ***6***

#### 2.3.1. Absorbance

Sensors **1** (1 mM) and **6** (1 mM) were dissolved in 0.1% DMSO/water for **1**, 2% DMSO/water for **6** and in 100% acetonitrile separately. The samples were left in the dark at an ambient temperature for 30 min. Absorbance of each sample was measured on the plate reader (λ = 300–700 nm).

Sensor **1** (1 mM) was then mixed with GSH (5 mM) and the absorbance was similarly measured. This mixture was then incubated in the dark overnight with the absorbance measured again. The experiments were carried out in triplicate.

#### 2.3.2. In-Solution Photoswitching of Sensor **1** in the Presence and Absence of GSH

A solution of **1** in 0.1% DMSO in water (50 µM, 100 µL) was incubated at an ambient temperature for 30 min. UV-Vis absorption (λ = 300–700 nm) of this sample was measured on the plate reader. The sample was mixed with GSH (5 mM) and incubated for 30 min at an ambient temperature followed by the measurement of UV-Vis absorption. This mixture was then exposed to UV light (365 nm) for 10 min, incubated in the dark for 30 min and re-exposed to UV irradiation for 10 min with UV-Vis absorption measured immediately after each step. The experiment was carried out in triplicate in the dark.

#### 2.3.3. Determining the Rate Constant and Half-Life of Sensor **1** upon Reaction with GSH

Sensor **1** (50 μM) in 0.1% DMSO in water was incubated at an ambient temperature for 30 min. UV-Vis absorption (λ = 300–700 nm) of this sample was measured on the plate reader. The sample was then mixed with GSH (5 mM) and the absorption of the sample was measured immediately after mixing. The absorption of this sample was monitored continuously for the next 120 s with an interval of 2 s between each read. The values were normalised to the absorbance of the sample without GSH using Microsoft Excel and plotted against time (s) in GraphPad Prism 7.0. A single exponential decay curve was fitted to the plot to extrapolate K_obs_ and t_1/2_.

#### 2.3.4. Fluorescence of Sensor **1** and **6** with Increasing Concentrations of GSH

Sensors **1** and **6** (50 μM) were separately incubated in 0.1% DMSO in water for 30 min at an ambient temperature. The solutions were then separately mixed with varying GSH concentrations ([GSH] = 0–5 mM). All concentrations of sensors **1** and **6** and GSH reported are final concentrations of the solution after mixing. The resultant fluorescence (λ_ex_ = 532 nm) spectra of **1** of each concentration of GSH was recorded on the plate reader (Figure S3A). Fluorescence was similarly measured for **6** with λ_ex_ = 478 nm (Figure S3B). The experiments were carried out in triplicate in the dark. The fluorescence intensities at 650 nm in Figure S3A were plotted against a log of GSH concentration in M to produce a standard curve of calibration for sensor 1. A linear trendline was fitted to the plot by GraphPad Prism 7.0.

#### 2.3.5. Quantum Yield Calculation

In a 96-well plate, the UV-vis absorbance and fluorescence spectra (λ_ex_ = 512 nm) of the solvent background (water for Rhodamine B, 0.1% DMSO for **1**) were recorded. This was repeated for five solutions with increasing concentration of **1** (25, 50, 75, 150, 250 μM) at excitation wavelength of 512 nm. This was repeated for Rhodamine B with concentrations of 0.1, 0.2, 0.5, 1, and 2 µM. The experiment was carried out in duplicate. A graph of integrated fluorescence vs. absorbance (Figure S4) was obtained. Quantum yield was calculated using the following equation:

Φ_x_ = Φ_ST_ (Grad_x_/Grad_ST_)(η^2^_x_/η^2^_ST_)

where subscripts ST and x denote standard (Rhodamine B) and **1**, respectively, Φ is fluorescence quantum yield, Grad is the gradient from the plot of integrated fluorescence intensity vs. absorbance and η is the refractive index of the solvent (0.1% DMSO in water = 1.3542 [32], water = 1.3332 [32]). The fluorescence quantum yield of rhodamine B in water at λ_ex_ = 512 nm is 0.3 as reported in literature [33].

#### 2.3.6. Selectivity of Sensor **1**

In a black 96-well plate, sensor **1** (50 µM) was separately mixed with solutions (1 mM) of various competing substrates (GSSG/Cys/Glu/Asp/2-mercaptaethanol/Ca^2+^/Zn^2+^/Na^+^/K^+^) in 0.1% DMSO in H_2_O. The mixtures were incubated in the dark for 30 min before fluorescence spectra (λ_ex_ = 532 nm) of each mixture was measured on the plate reader. Premixed samples containing **1** (50 µM), a competing substrate (1 mM), and GSH (5 mM) were incubated in the dark at an ambient temperature for 30 min. Fluorescence spectra of each sample was similarly measured. The experiments were carried out in duplicate.

### 2.4. Cell-Based Experiments

#### 2.4.1. Cell Viability Assay

Chemical toxicity was quantified using the AlamarBlue assay (Molecular Probes, Eugene, OR, USA), as previously described [34]. HEK293T cells were plated a 1 × 10^4^ cells/well in 96-well plates, and the fluorescence were measured with a FLUOstar Optima microplate reader (BMG Labtech, Ortenberg, Germany) after 21 h of incubation with sensor **1** (50 µM) to obtain the quantitative measures of cell viability. Cells were incubated with HgCl (25 µM) for 1 h to generate the positive control for cytotoxicity.

#### 2.4.2. Confocal Cell Imaging

HEK293T cells were cultured in DMEM medium (Thermo Fisher, Waltham, MA, USA) containing 10% heat inactivated fetal bovine serum (FBS) (Thermo Fisher, Waltham, MA, USA), 1% penicillin-streptomycin (Sigma, St. Louis, MO, USA), 1% L-glutamine (Thermo Fisher, Waltham, MA, USA), and 2‰ fungizone (Thermo Fisher, Waltham, MA, USA). Cells were plated at 2.7 × 10^5^ cells/mL density in 8 well ibidi-slide (ibidi, Planegg, Germany). Cell were incubated for 4 h in medium with or without 10 mM buthionine sulphoximine (BSO, Sigma, St. Louis, MO, USA). Cells were incubated for 15 min in PBS containing 50 µM sensor **1**, then washed 3 times using warm PBS saline. Plates were exposured under a UV lamp for 10 min to activate 1 prior to imaging. An Olympus FluroView V10i confocal microscope was used for all the imaging. For the imaging of cells treated with GSH sensor, λ_Abs_/λ_Em_ = 559/570–670 nm was used. Same laser intensities and reading sensitivities were used for all imaging sessions. Cell images were processed and the overall fluorescence intensity of each sample was measured using ImageJ Fiji.

## 3. Results and Discussion

### 3.1. Design and Synthesis of Sensor ***1***

Sensor **1** is somewhat unique amongst spiropyrans in that it exists predominantly as the more fluorescent MC isomer in an aqueous environment. Fluorescence is ‘turned-off’ on reaction with GSH with formation of the SP isomer, which has significantly weaker fluorescence (see Figure 1, **1-SP**). Sensor **1** would be expected to exist in its ‘off state’ in the presence of millimolar concentrations of GSH, as found in healthy cells. Reduced GSH levels would then shift the equilibrium to the MC isomer (Figure 1, **1-MC**), resulting in a higher fluorescence and detection.

A 6-nitro substituent was incorporated onto the component spiropyrans of **1** to promote the formation of the MC isomer under aqueous conditions [30,35]. In addition, a more sterically demanding dimethyl-piperazine core was incorporated into the design to suppress non-covalent binding of GSH to the MC isomer as was proposed for the literature spiropyran-based ‘turn on’ sensor [26]. Finally, a 5′-carboxylate group was introduced to enhance hydrophilicity to further improve aqueous solubility and compatibility over the earlier sensor [28].

**Figure 2:**
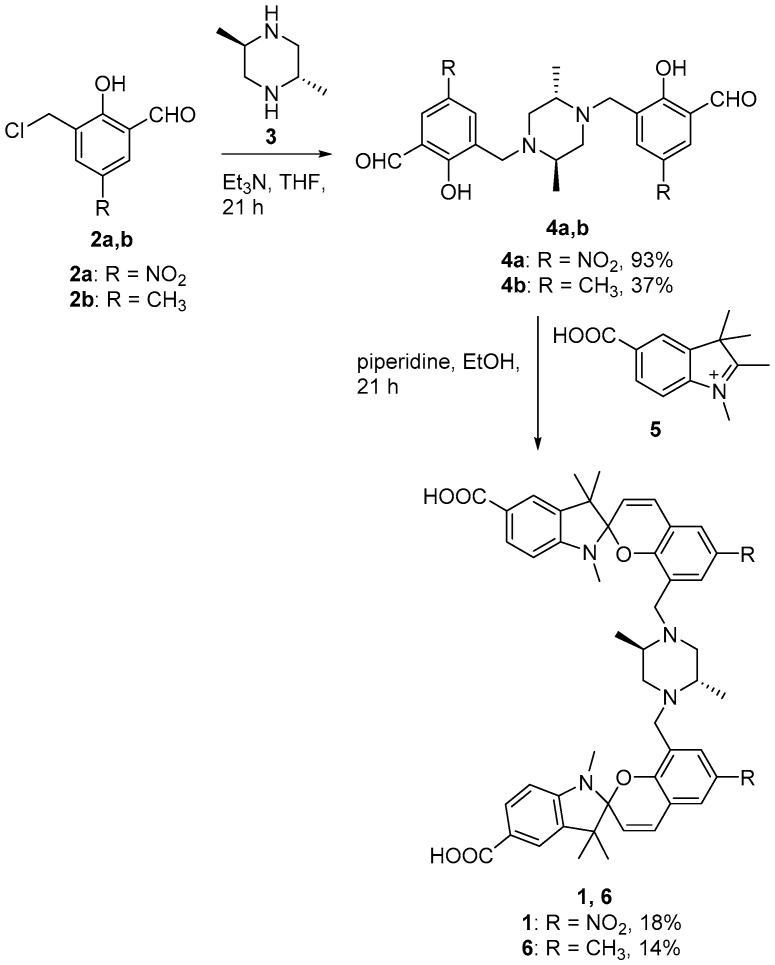
Synthesis of analogues **1** and **6**.

The synthesis of sensor **1** is outlined in Figure 2. Briefly, base-catalyzed Mannich-type reaction of hydroxybenzaldehyde **2a** with trans-2,5-dimethylpiperazine (**3**) gave the key bis-adduct **4a** in approximately 93% yield. Subsequent piperidine-catalyzed condensation of **4a** with indolenine **5** gave **1** that was purified by C18 reverse phase HPLC. The methyl analogue of **1** (compound **6**) was similarly synthesised using starting materials **4b** and **5**.

### 3.2. Spectroscopic Analysis of Sensor ***1***

**Figure 3:**
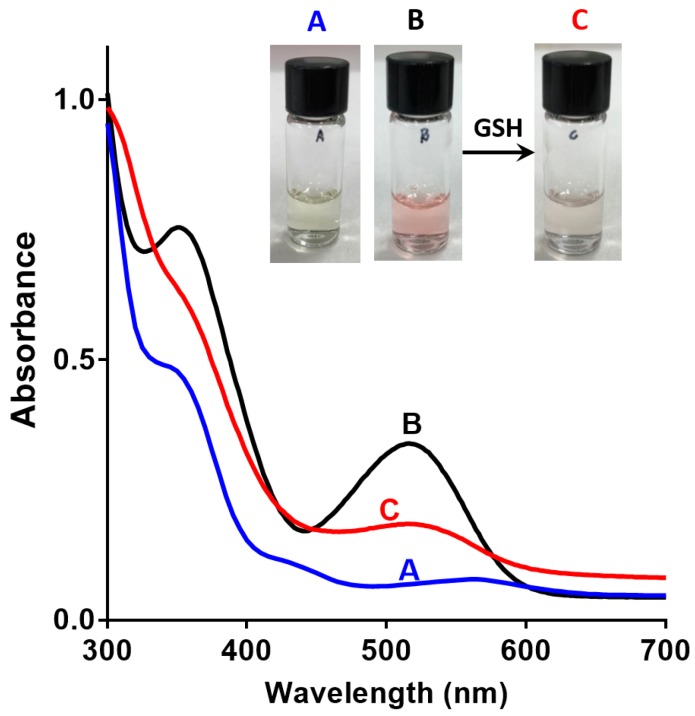
Absorption spectra and picture of (**A**) Sensor **1** in acetonitrile (1 mM); (**B**) Sensor **1** in 0.1% DMSO in water (1 mM); (**C**) Sensor **1** (1 mM); and, GSH (5 mM) in 0.1% DMSO in water.

The solubility and spectroscopic properties of sensor **1** were investigated. Sensor **1** is readily soluble in 0.1% DMSO in water, a solvent mixture that is compatible [36] with most biological applications. This represents a significant improvement on the previously reported ‘turn-on’ spiropyran-based sensor of GSH, which displays a marginal solubility in 10% ethanol in water [26]. The aqueous solubility of **1** is also superior to that of monochlorobimane (mCB) [14], a commonly used fluorescent reaction-based chemosensor for GSH, which requires high concentrations of organic solvents such as DMSO and ethanol for sensing applications. Next, the base-line SP/MC composition of **1**, in the absence of GSH, was defined by UV-vis absorption spectroscopy to confirm the design, and the results are shown in Figure 3A,B. A solution of sensor **1** in 0.1% DMSO/water (1 mM) exhibited an absorbance in the region of 512–522 nm in the absence of GSH, which is characteristic of the colored ring opened MC isomer (see Figure 3B). A non-aqueous solution of **1** in acetonitrile (1 mM, Figure 3A) showed no such absorption peak, a result consistent with the ring-closed and less colored SP isomer in this case. This is a critical finding for a ‘turn-off’ sensor, where the ring-opened fluorescent MC isomer must predominate in the absence of analyte (GSH) in a polar protic solvent system. Interestingly an analog of **1**, with methyl groups in place of the nitro groups, existed predominantly as the SP isomer in both aqueous and acetonitrile solvent systems, as seen in SI Figure S1.

**Figure 4:**
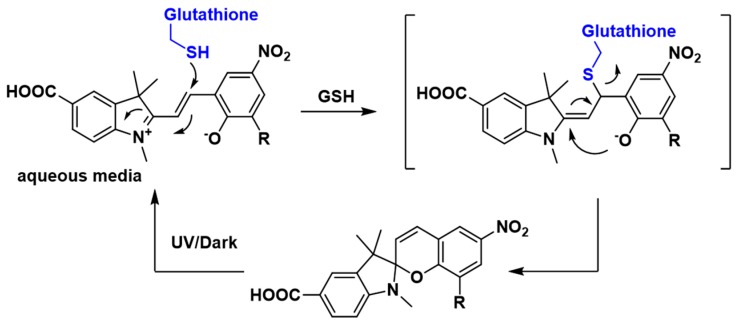
Possible mechanism for reversible binding of γ-Glutamyl-cysteinyl-glycine (GSH) to sensor **1**.

**Figure 5:**
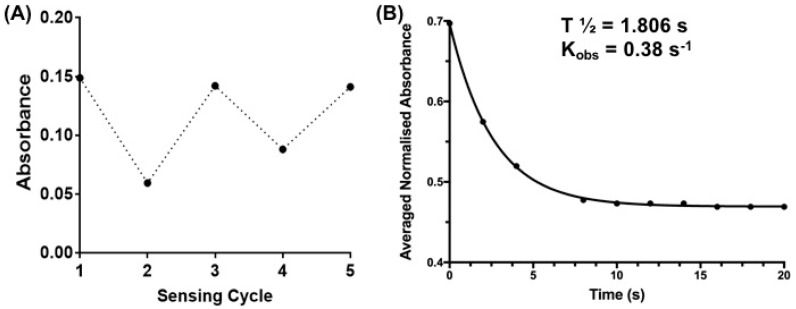
(**A**) Reversibility of sensor **1** (50 µM) at λ = 550 nm: Sensing cycle (1) Represents absorbance of sensor **1** in the absence of GSH; (2) and (4) represents the absorbance readings after addition of 5 mM GSH and sensing cycles (3) and (5) represent absorbance readings after exposure of sensor **1** + GSH (5 mM) to UV irradiation (λ = 365 nm) for 10 min; (**B**) Time-dependent absorbance decay curve of sensor **1** after the addition of GSH (5 mM, final concentration) in 0.1% DMSO in water. Recording interval: 2 s. Curve (R^2^ = 0.9996) obtained using GraphPad Prism 7.0 (one-phase decay). Insert shows the observed rate constant (K_obs_) and calculated half-time (t_1/2_).

Next, the reversibility of the sensor was studied in the presence of 5 mM GSH to ascertain if GSH binding and release can be controlled and defined by measuring the state-dependent absorbance. The addition of GSH to the DMSO/water solution of sensor **1** resulted in a decrease in absorbance in the above-mentioned 512–522 nm region (Figure 3C). This is consistent with the formation of the SP isomer, possibly by a conjugate addition of GSH to **1-MC** followed by cyclisation, as outlined in Figure 4. GSH is known to undergo conjugate addition, and this has been used as the basis of a number of sensors [11,13,37]. The solution was irradiated with UV light (λ = 365 nm) to switch the sensor back to the more colored MC isomer, with a re-appearance of the absorption in the region of 512–522 nm. Repeated switching between the SP and MC isomer with light of specific wavelength produced reproducible changes in absorbance intensities (see Figure 5A), which demonstrates that sensor **1** can be used for multiple measurements of GSH.

The time-dependent absorbance decay of sensor **1** in the presence of excess GSH was measured at room temperature in 0.1% DMSO/water to approximate the reaction rate for this reaction. The decrease in absorbance peak of the MC isomer (512–520 nm) was measured at 2 s intervals upon addition of GSH, and the results are shown in Figure 5B. The reaction is well approximated by a single exponential decay (‘turn-off’) to give an observed rate constant of 0.38 s^−1^ and half-life of 1.8 s (Figure 5B). The reaction was complete in under 5 s, a result compatible with live-cell imaging of GSH dynamics that require a temporal resolution of seconds. Further work is required to fully characterize the kinetics of the reaction, but it is important to note that the observed reaction rate between sensor **1** and GSH is within the time-scale of recently reported GSH ‘turn-on’ sensors [13,37,38].

**Figure 6:**
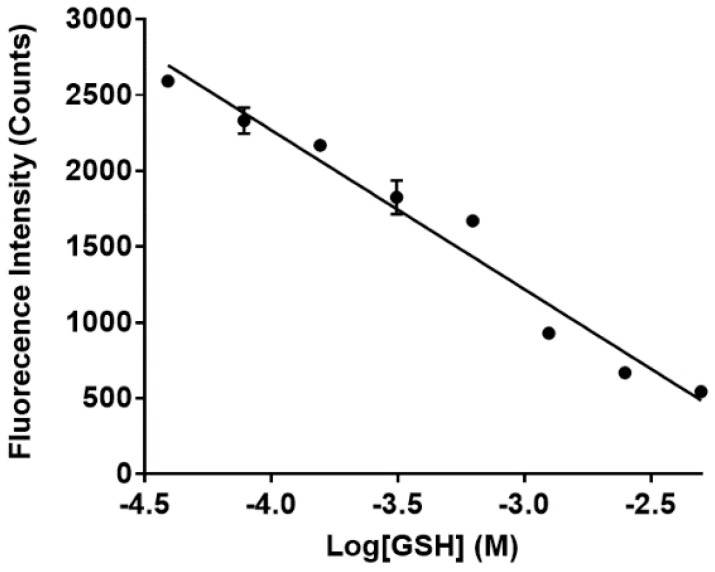
Fluorescence of sensor **1** (final concentration 50 µM) in the presence of GSH. Maximum fluorescence intensities of **1** (λ_em_ = 650 nm) in the presence of increasing concentration (0–5 mM) of GSH was plotted against the log of GSH concentrations in M.

The fluorescence of sensor **1** in the presence of GSH was also investigated to further extend its sensing capabilities, e.g., for application in live-cell imaging using confocal microscopy. An aqueous solution of sensor **1**, in the absence of GSH, gave a fluorescence emission maximum at approximately ~655 nm (λ_ex_ = 532 nm) which is consistent with the MC isomer based on the earlier studies (Figure S2A). This fluorescence then decreases linearly with increasing GSH concentrations as shown in Figure 6. Importantly, fluorescence emission for the 6-methyl analogue of **1** mentioned above was not observed in either the presence or the absence of excess GSH (Figure S2B). This compound exists as the SP isomer and clear does not react with GSH, as per the earlier absorbance results. The quantum yield of **1**, in the absence of GSH (0.1% DMSO in water), was determined to be 0.029 using rhodamine B as the calibration standard as detailed in the SI, Figure S3. This result is comparable to the previously reported spiropyran-based sensors [39,40].

**Figure 7:**
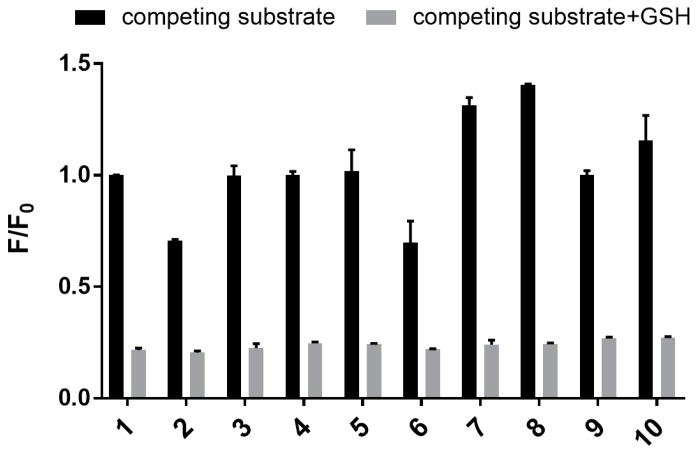
Bar graph showing fluorescence of **1** (50 µM) in the presence of a range of biologically relevant substrates: 1. blank; 2. glutathione disulfide (GSSG); 3. Cys; 4. Asp; 5. Glu; 6. 2-mecaptoethanol (BME); 7. Na^+^; 8. K^+^; 9. Ca^2+^; 10. Zn^2+^. Bars represent the fluorescence response with added GSH and/or substrates (*F*) over the response in the absence of added substrate and GSH (*F*_0_). Black bars represent samples in the absence of GSH and with added competing substrate; grey bars represent samples with both competing substrate and GSH. Excitation wavelength = 532 nm, emission wavelength = 650 nm.

Next, sensor **1** was assayed against a range of biologically relevant metal ions (Na^+^, K^+^, Ca^2+^, Zn^2+^), amino acids (Cys, Asp, Glu), glutathione disulfide (GSSG), and 2-mecaptoethanol (BME) to define its selectivity profile. The sensor clearly shows good selectivity for GSH, with only GSSG and BME showing any (weak) affinity (see Figure 7, black bars). Similar selectivity has been reported for other mechanism-based GSH sensors [15,37]. Interestingly, sensor **1** displayed an excellent selectivity for GSH over GSSG. The sensor also has a reduced affinity for BME. In both cases this may reflect reduced nucleophilicity [41,42]. To our delight, the sensor showed no affinity toward cysteine, an observation consistent with other reported GSH mechanistic sensors [11,13,37]. Competition assays were also carried out to determine the selectivity of sensor **1** for GSH in the presence of a competing analyte. In a 96-well plate, separate solutions of sensor **1** and GSH (5 mM) were incubated with each of the abovementioned analytes for 30 min, and the resulting fluorescence emission was recorded. The results shown in Figure 7 (see grey bars) clearly show that sensor **1** has excellent selectivity for GSH over other biologically related analytes in co-solution.

### 3.3. Sensing of Intracellular GSH Using Sensor ***1***

**Figure 8:**
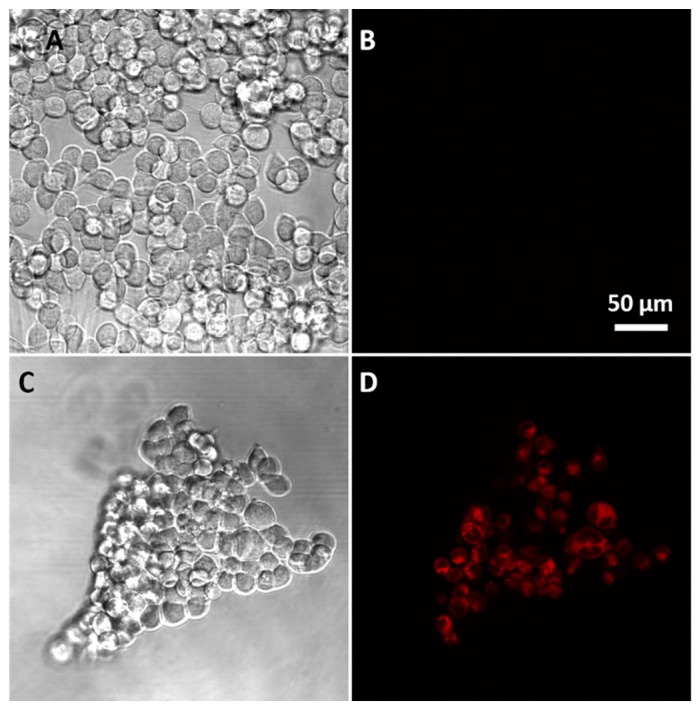
Confocal microscopic images of HEK293 cells (**A**) without the addition of sensor **1**; **(B**) bright-field image of (**A**); (**C**) incubated with sensor **1** (50 µM) in PBS buffer for 15 min; and, (**D**) bright-field image of (**C**).

**Figure 9:**
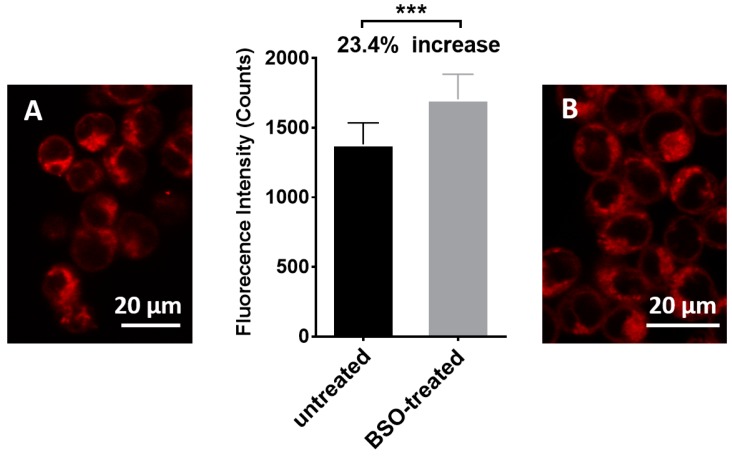
Relative fluorescence intensity of untreated cells and BSO treated cells. The fluorescence of nine small random areas in a cell image were measured for each treatment using ImageJ Fiji. Unpaired student *t*-test was used with *p* < 0.05 (***). (**A**): confocal microscopic image of HEK293 cells incubated with **1**; (**B**): confocal microscopic image of HEK293 cells pre-treated with BSO and incubated with **1**.

Finally, the ability of sensor **1** to detect different levels of intracellular GSH was examined in HEK293 cells, a good model for this experiment as the cell line has been extensively used in previous sensing studies [43,44,45,46,47]. HEK293 cells were treated with a potent and specific inhibitor of glutathione synthesis (buthionine sulphoximine, BSO) for 4 h^33^ to lower intracellular levels of GSH. Treated and untreated cells were then separately incubated with sensor **1** for 15 min, with final imaging on a confocal fluorescence microscope using the green laser for excitation. The resulting fluorescence images (Figure 8B,D and Figure 9) reveal stronger red fluorescence within the BSO treated cells characteristics of the MC isomer (see Figure 1). Such red fluorescence is advantageous for biological imaging since auto-fluorescence and scattering within tissues is minimal at these longer wavelengths. Incubating the cells with sensor **1** (50 µM) for 21 h at 37 °C, an incubation time that exceeds the required experimental time, revealed in that sensor **1** had no effect on the cell viability and is non-toxic under the experimental conditions (See SI, Figure S4). The findings are supported by previous studies where micromolar concentrations of spiropyran derivatives were reported to have minimal cytotoxicity in macrophages, gastric cells, and epithelial cells even after 72 h of exposure [48]. Sensor **1** is clearly cell permeable and can detect intracellular GSH (Figure 8) with fluorescence intensity inversely proportional to the concentration of GSH, as was observed in the solution-based experiments. The difference in fluorescence intensity was observed for untreated and BSO-treated cells (~24%, Figure 9) is consistent with a reported ~30% decrease in GSH levels in the presence of BSO [49]. This is an important finding as it demonstrates the potential of sensor **1** to detect and quantify the decreases in intracellular GSH. Future work is focused on using the sensor to examine intracellular levels of GSH in systems with endogenously depleted glutathione such as cancer cells.

## 4. Conclusions

In summary, we present, to the best of our knowledge, the first reversible ‘turn-off’ sensor **1** for γ-Glutamyl-cysteinyl-glycine (GSH). Sensor **1** is unique in that it exists as the highly fluorescent MC isomer in the aqueous environment. The addition of GSH results in a decrease in both the fluorescence and the emission of sensor **1**, indicating the formation of the SP isomer possibly through a reversible conjugate addition and cyclisation sequence. **1-MC** is regenerated on UV irradiation to enable multiple cycles of GSH sensing. Sensor **1** is soluble in aqueous media and displays an excellent selectivity for GSH over other biologically related analytes, importantly including other thiols. Sensor **1** can detect changes of intracellular GSH in live HEK293 cells to provide a potentially regenerable sensor for monitoring lower levels of intracellular GSH as associated with the onset of important diseases. Future work will be focused on using the sensor to quantify GSH in degenerate human umbilical vein endothelial cells (HUVEC) and in mouse oocyte, to advance our understanding of oxidative damage in these cell types.

## Supplementary Materials

The following are available online at http://www.mdpi.com/2079-6374/7/3/36/s1, NMR spectra, Figure S1: Absorbance of sensor **6** (1 mM) in 2% DMSO in water (black) and acetonitrile (blue), Figure S2: Fluorescence of Sensor **1** and **6**, Figure S3: Quantum yield of sensor **1**, Figure S4: Cytotoxicity of Sensor **1**.
