# Supplementary material for: A Rationally Designed Reversible ‘Turn-Off’ Sensor for Glutathione
Source: Biosensors (Basel). 2017 Sep 6;7(3):36. doi: 10.3390/bios7030036 (PMC5618042; doi:10.3390/bios7030036)
Supplement: Supplementary file 1 [file biosensors-07-00036-s001.pdf]

A Rationally Designed Reversible 'Turn-Off' Sensor for Glutathione

Sabrina Heng*[1], Xiaozhou Zhang*[1], Jinxin Pei[2], Andrew D. Abell[1]

*equal first authors

[1] ARC Centre of Excellence for Nanoscale BioPhotonics, Institute of Photonics and Advanced Sensing, Department of Chemistry, School of Physical Sciences, The University of Adelaide, South Australia, 5005.

[2] Discipline of Physiology, Faculty of Health Sciences, The University of Adelaide, South Australia, 5005.

E-mail: sabrina.heng@adelaide.edu.au, andrew.abell@adelaide.edu.au

NMR Spectra

^{1}H NMR for compound **4a**.

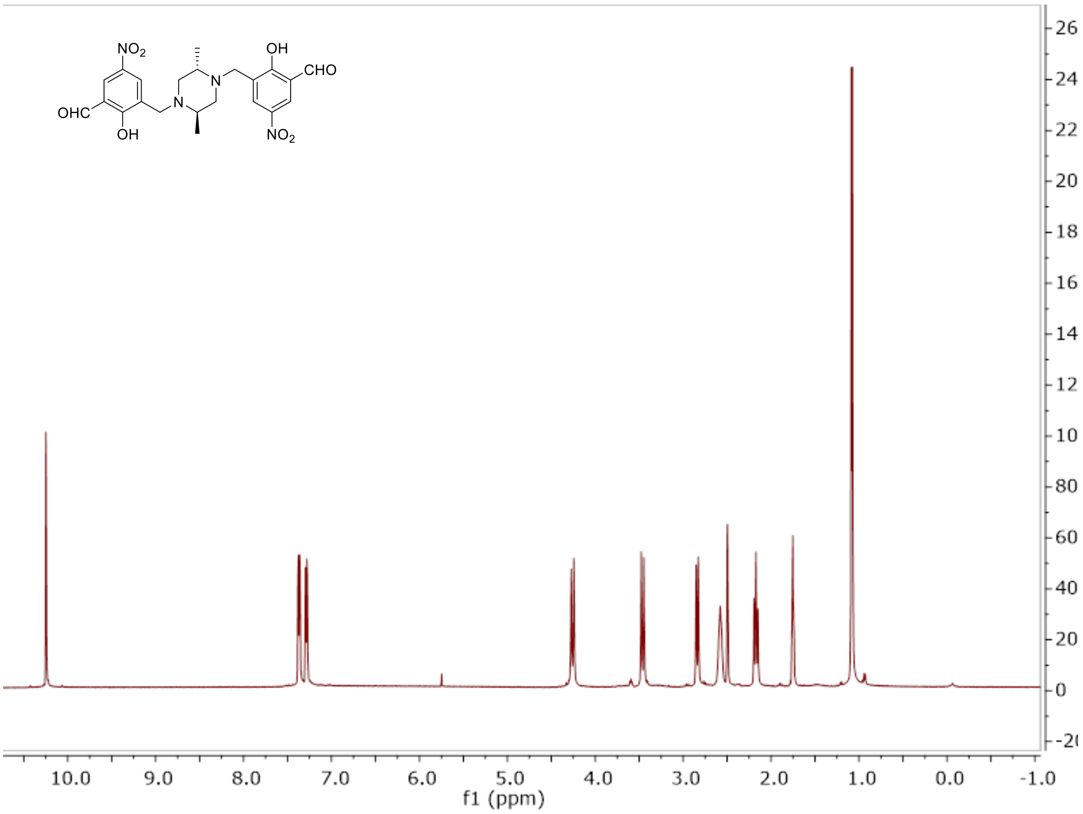

^{13}C NMR for compound **4a**.

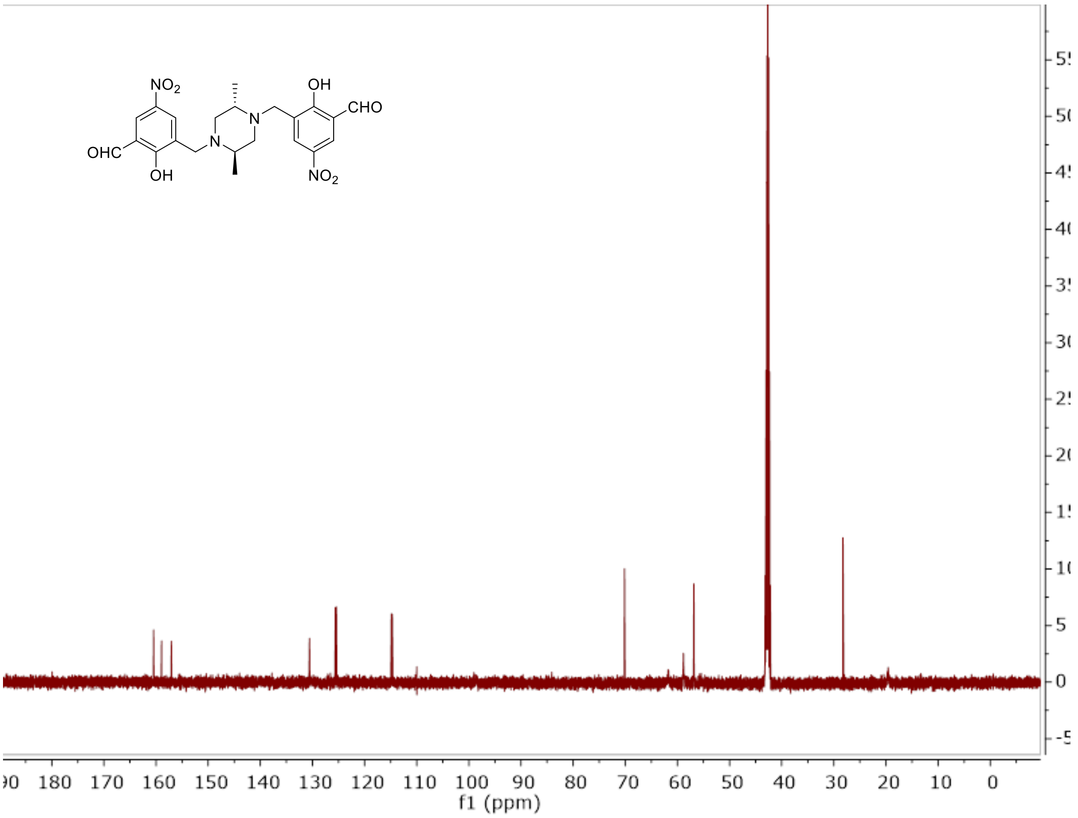

^{1}H NMR of compound **4b**.

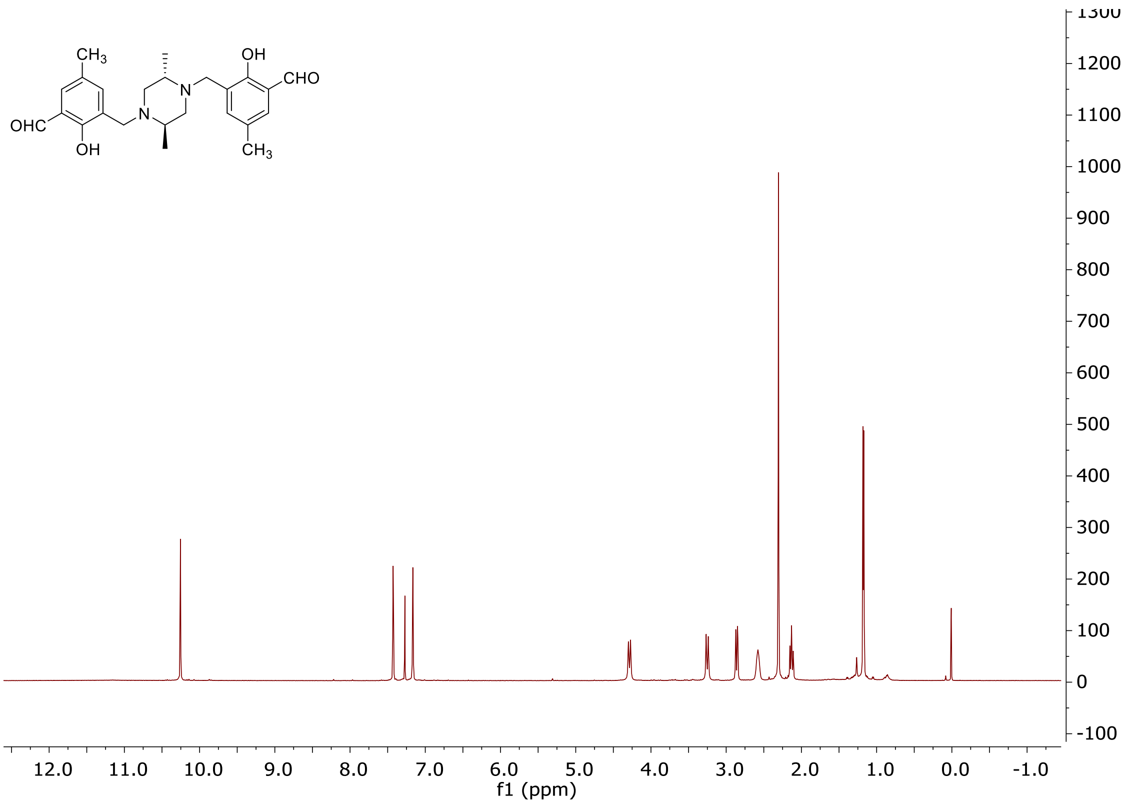

^{13}C NMR of compound **4b**.

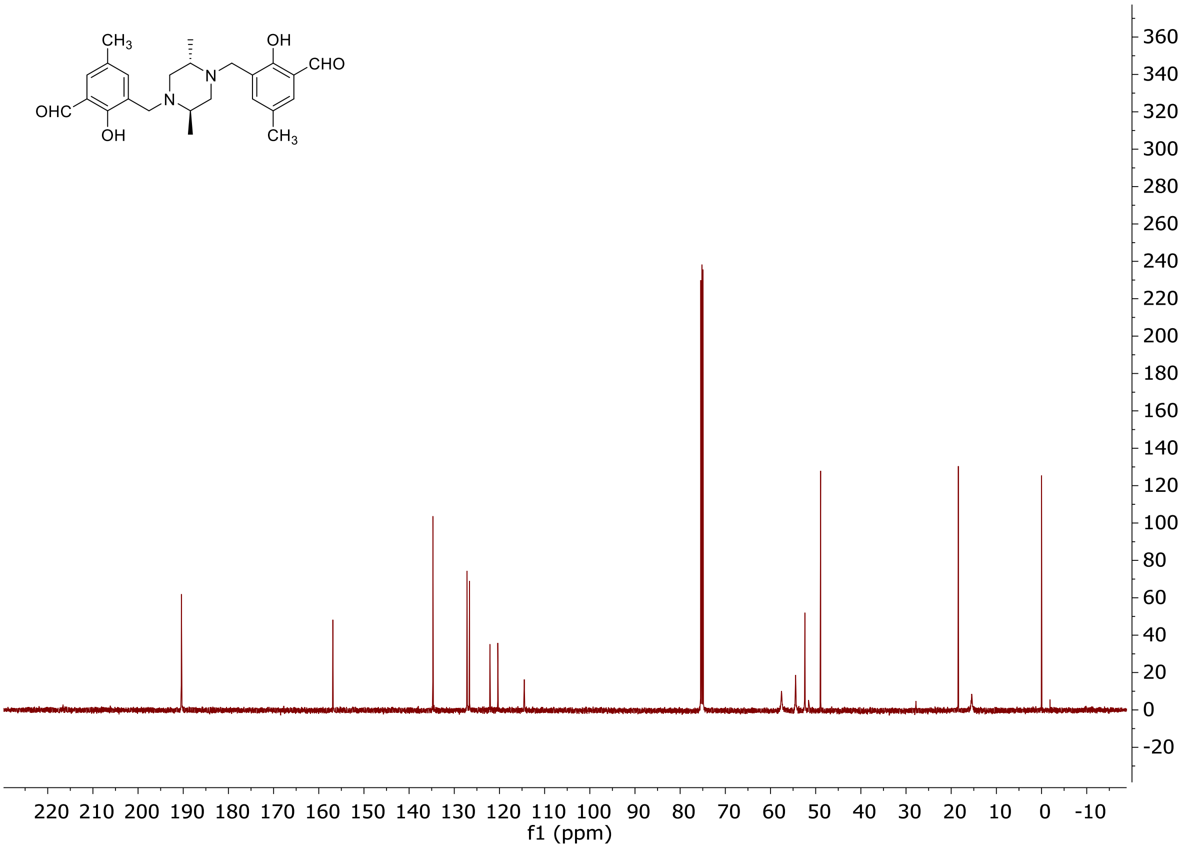

^{1}H NMR for compound **1**.

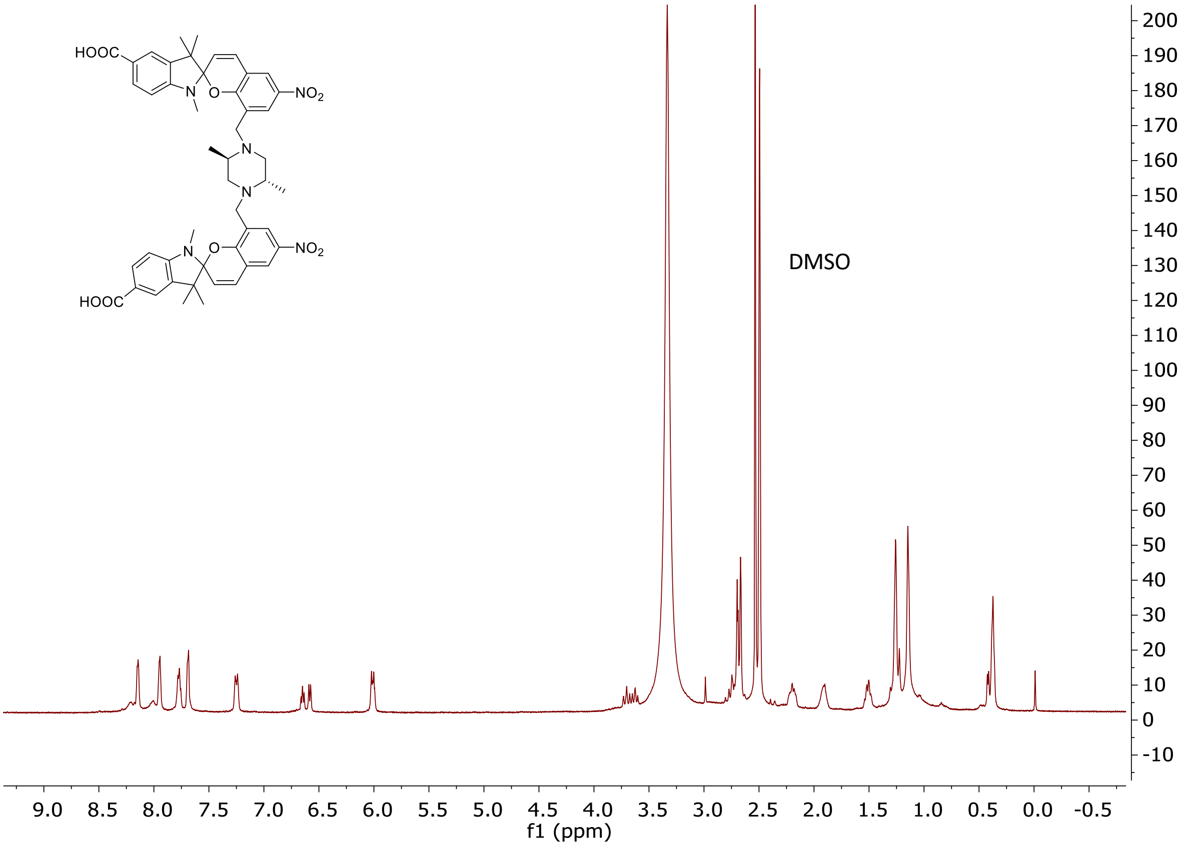

^{13}C NMR for compound **1**.

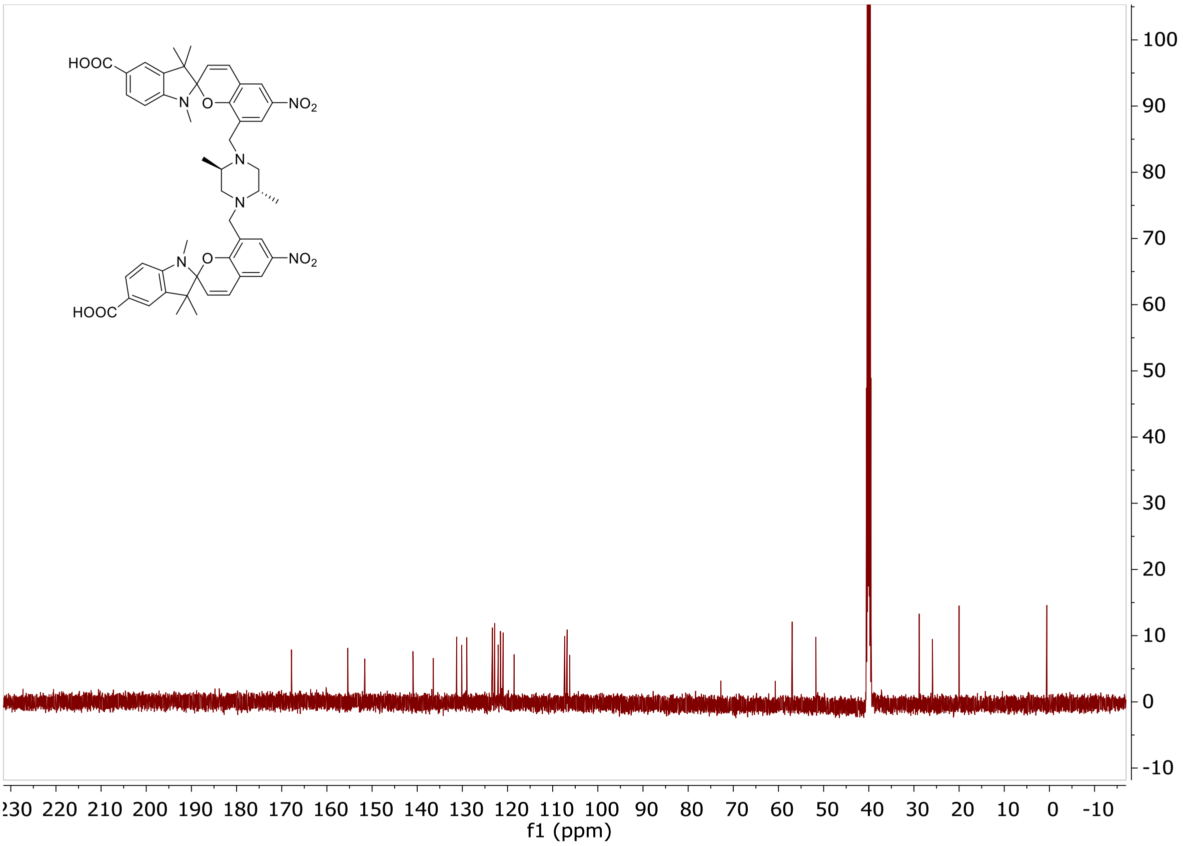

^{1}H NMR of compound **6**.

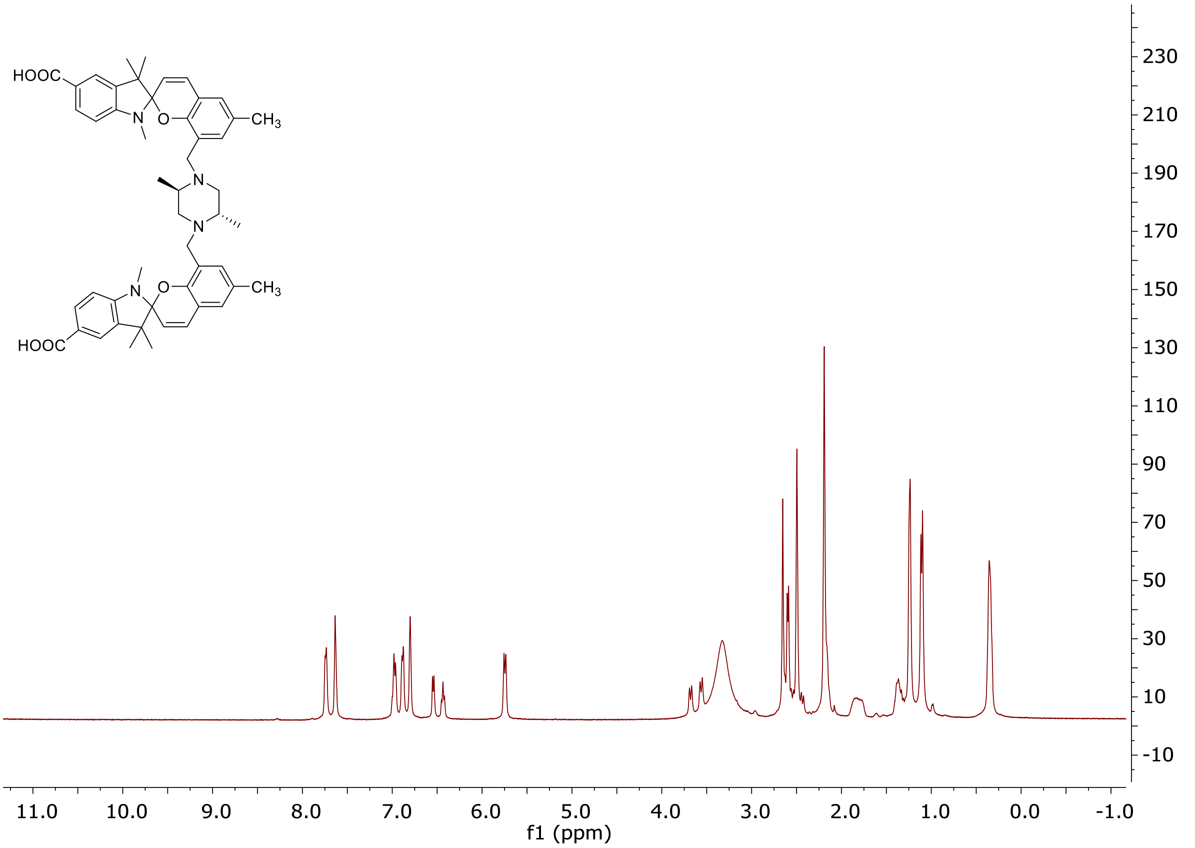

^{13}C NMR of compound **6**.

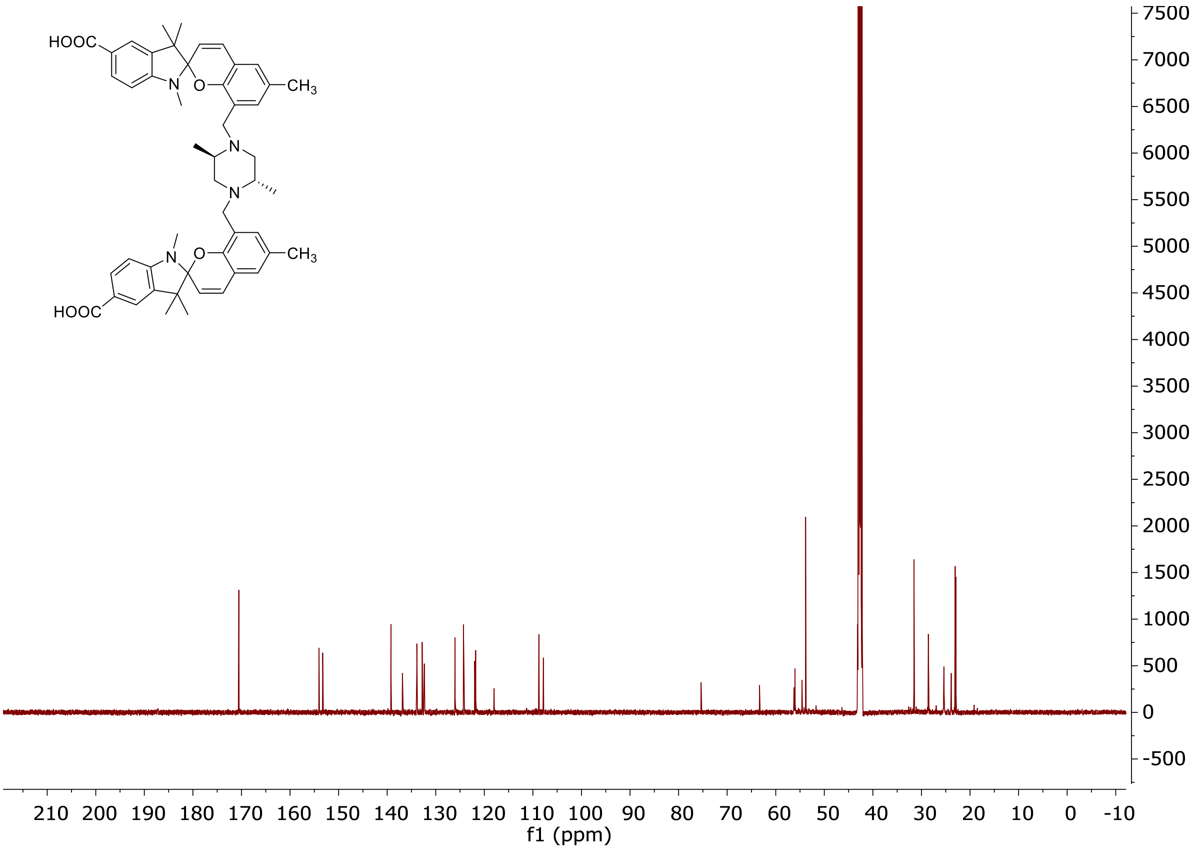

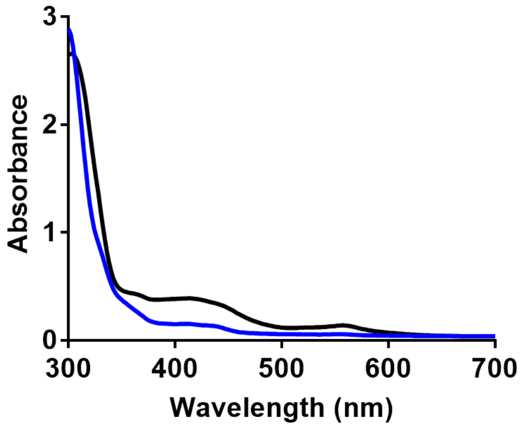

Figure. S1. Absorbance of sensor **6** (1 mM) in 2% DMSO in water (black) and acetonitrile (blue).

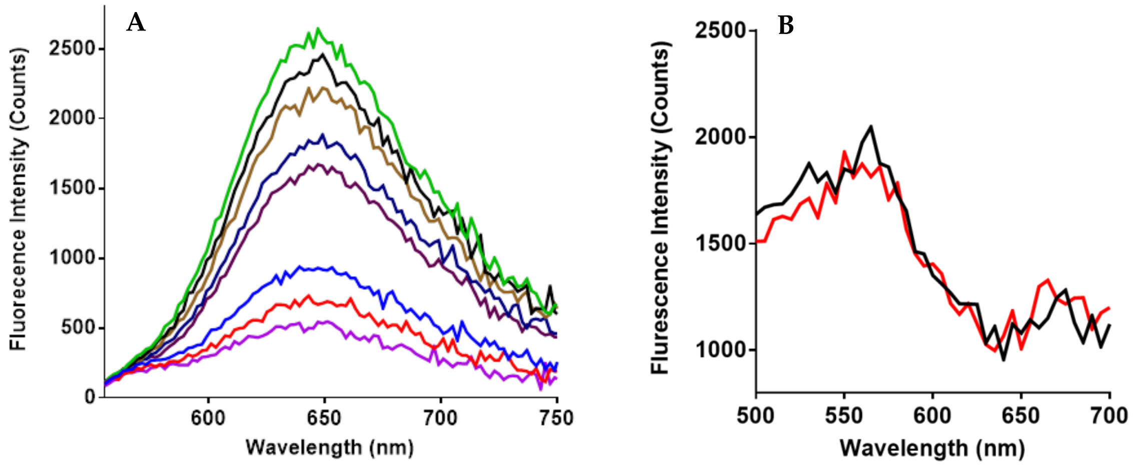

Figure. S2. A. Fluorescence spectra of sensor **1** (final concentration = 50 μM) obtained after 30 min incubation with 0 — ; 78 μM —; 156 μM — ; 313 μM — ; 625 μM — ; 1.25 mM — ; 2.5 mM — ; and 5 mM — GSH in 0.1 % DMSO in water. λ_{ex} = 532 nm. B. Fluorescence spectra of **6** (final concentration = 50 μM) obtained after 30 min incubation with 0 — and 5 mM — GSH.

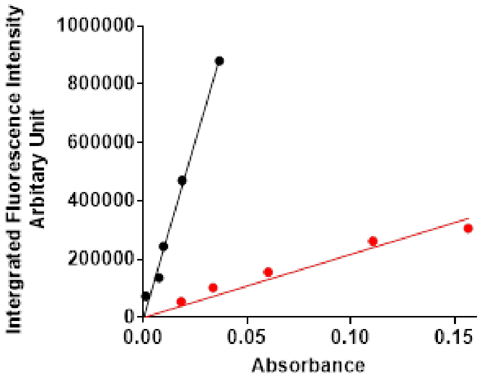

Figure. S3. Linear plots for rhodamine B (black) and sensor **1** (red) respectively. The gradient for each sample is proportional to that sample's fluorescence quantum yield.

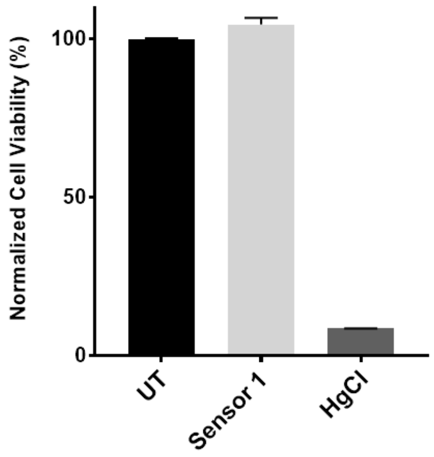

Figure. S4. Viability of untreated HEK293T cells (black), cells incubated with sensor **1** (light grey) for 21 h at 37 ^{o}C and cells incubated with HgCl for 21 h at 37 ^{o}C (dark grey).
